# Protein Loosing Enteropathy Secondary to Strongyloidiasis: Case Report and Review of the Literature

**DOI:** 10.1155/2016/6831854

**Published:** 2016-01-06

**Authors:** Weam El Hajj, Gilbert Nakad, Antoine Abou Rached

**Affiliations:** ^1^Department of Internal Medicine, Gastroenterology Division, Faculty of Medical Sciences, Lebanese University, Hadath Campus, P.O. Box 3, Hadath, Beirut 2903 1308, Lebanon; ^2^Department of General Surgery, Faculty of Medical Sciences, Lebanese University, Hadath Campus, P.O. Box 3, Hadath, Beirut 2903 1308, Lebanon

## Abstract

Strongyloidiasis is a helminthic disease which affects millions around the world resulting in a significant burden in certain high risk groups. It is rarely reported in the Lebanese population probably due to the low index of suspicion in common practice. We are reporting a case of strongyloidiasis that was found in an elderly patient presenting initially with dyspnea followed by skin rash, protein loosing enteropathy, diarrhea, and abdominal pain while on corticosteroid therapy. The diagnosis was suspected based on clinical presentation in addition to peripheral eosinophilia. We will also describe the upper and lower endoscopic aspects of the disease, as well as histologic findings on duodenal and colonic biopsies.

## 1. Introduction

Intestinal nematode infections are frequently encountered worldwide with a net predominance in the hot and humid tropical and subtropical areas. Strongyloidiasis is among the most important helminthic infections affecting humans and it may manifest clinically as mild or asymptomatic disease persisting for decades, but also it can have life-threatening manifestations. In Lebanon, it is an unusual infection and is not frequently reported in the literature. We are hereby reporting a case of strongyloidiasis in a Lebanese patient presenting in a picture of diarrhea and refractory hypoalbuminemia after two months' history of unexplained dyspnea and skin rash.

## 2. Case Presentation

This is a case of 77-year-old male patient who presented for lower limbs edema and abdominal pain with diarrhea of 1-month duration.

The patient was an ex-smoker and nonalcoholic. His past surgical history included cholecystectomy and prostatectomy. His medical history was significant for hypertension controlled on Bisoprolol and Irbesartan, dyslipidemia treated with Atorvastatin, and a transient cerebral ischemic attack 5 months prior to presentation for which he was put on aspirin and clopidogrel. At that time, he was incidentally found to have peripheral eosinophilia of 1050 eos/mm^3^ (13.7% of total leukocytes) without going into further investigations. As family history, he had a brother diagnosed with Non-Hodgkin Lymphoma who died few months earlier after a prolonged hospitalization for unexplained dyspnea, attributed later on to a* Strongyloides stercoralis* infection detected in his bronchoalveolar lavage only one day before his death.

**Figure 1:**
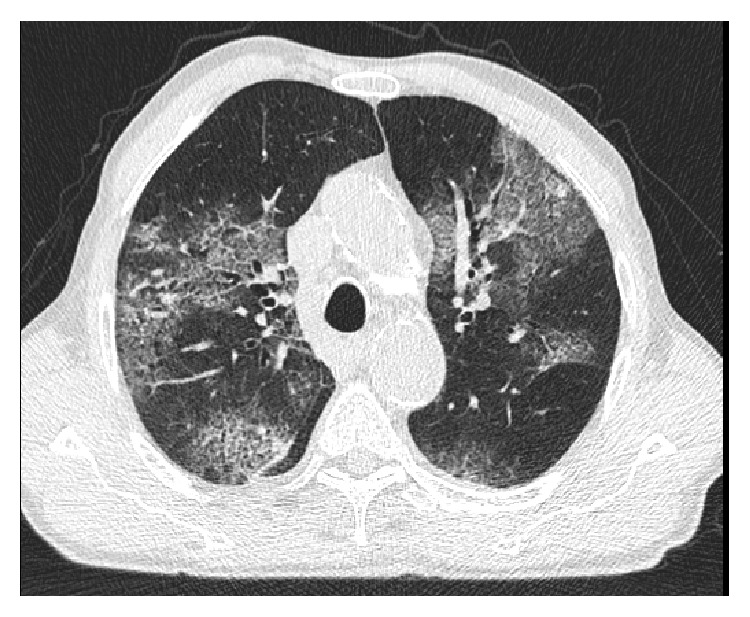
Chest CT scan done during the first admission showing bilateral patchy asymmetric interstitial lung disease suspicious of allergic alveolitis or eosinophilic pneumonitis.

His history went back to 3 months prior to presentation when he was admitted to another hospital for dyspnea, cough, and high grade fever that started few days after inadvertent inhalation of pesticides while working in his garden. CT chest was done and showed bilateral patchy interstitial infiltrates suspicious of allergic pneumonitis (Figure 1). Bronchoscopy was also done and showed normal bronchial mucosa. Bronchoalveolar lavage (BAL) analysis showed an inflammatory smear and the culture detected few Alcaligenes spp. Blood culture was negative. So patient was considered having pulmonary infection on top of allergic pneumonitis and was discharged home on levofloxacin and prednisone 1 mg/kg for 1 week to be tapered down over 3 weeks thereafter. Although his dyspnea and chest infiltrates were improving during steroids tapering, our patient developed severe diffuse pruritic skin rash that persisted for about 10 days and he started experiencing a progressively increasing epigastric discomfort associated with diarrhea. In addition, he noted an increasing bilateral lower limbs edema. After stopping steroids, his skin rash disappeared but without improvement of other symptoms.

His diarrhea was watery and nonmucoid accompanied sometimes by fine streaks of blood and having a variable frequency between 1 and 7 episodes per day. It was associated with moderate to severe left lower quadrant abdominal pain not related to food, as well as epigastric pain and many episodes of postprandial vomiting. He also reported a decreased PO intake and a significant weight loss of 12 kg over the last 2 months. His lower limbs edema was progressively increasing, even after stopping steroids. He had no significant dyspnea or cough and no urinary symptoms.

On physical examination, the patient's heart rate was 80 beats per minute, blood pressure was 110/70 mmHg, and temperature was 37°C. He was looking ill without scleral icterus or palpable cervical lymph nodes. His chest auscultation was unremarkable. His abdomen was soft with moderate epigastric and left lower quadrant tenderness and normal bowel sounds. Lower limbs examination showed severe 4+ pitting edema extending from the ankles till the knees. There were no skin rashes and the neurological exam was unremarkable.

Initial tests showed a white blood cell count of 8200 per mm^3^ with 14% eosinophils, hemoglobin around 10 g/dL, MCV around 84 fL, creatinine level around 2 mg/dL, and sodium level of 127 meq/L, total serum proteins were 4.5 g/dL with albumin of 1.8 g/dL, liver enzymes were normal, and International Normalized Ratio (INR) was slightly prolonged (1.5). Direct fecal examination was done once and showed no parasites. Stools culture turned out to be negative. Chest X-ray was within normal limits. Twenty-four-hour urine collection revealed only 180 mg of proteins.

**Figure 2:**
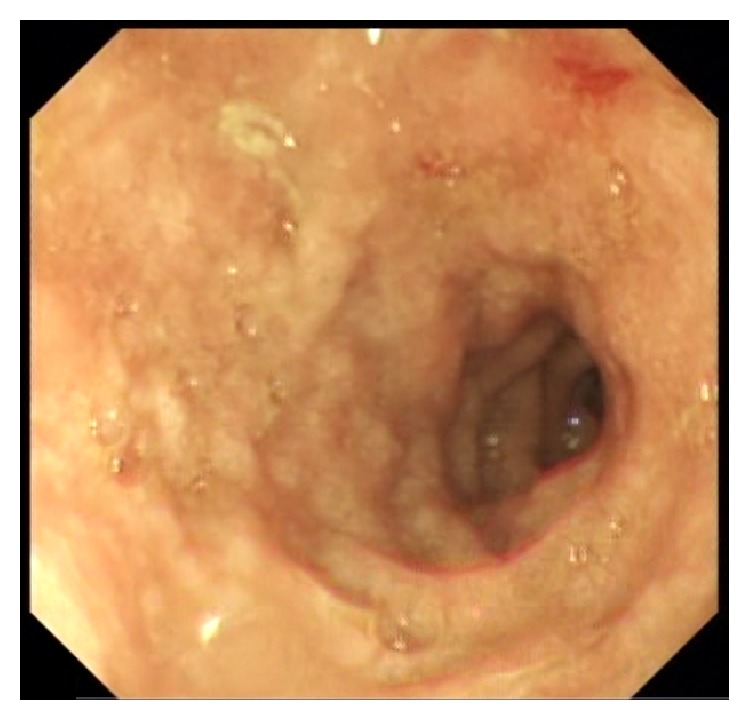
Duodenal bulb showing edematous nodular mucosa with erythematous spot.

**Figure 3:**
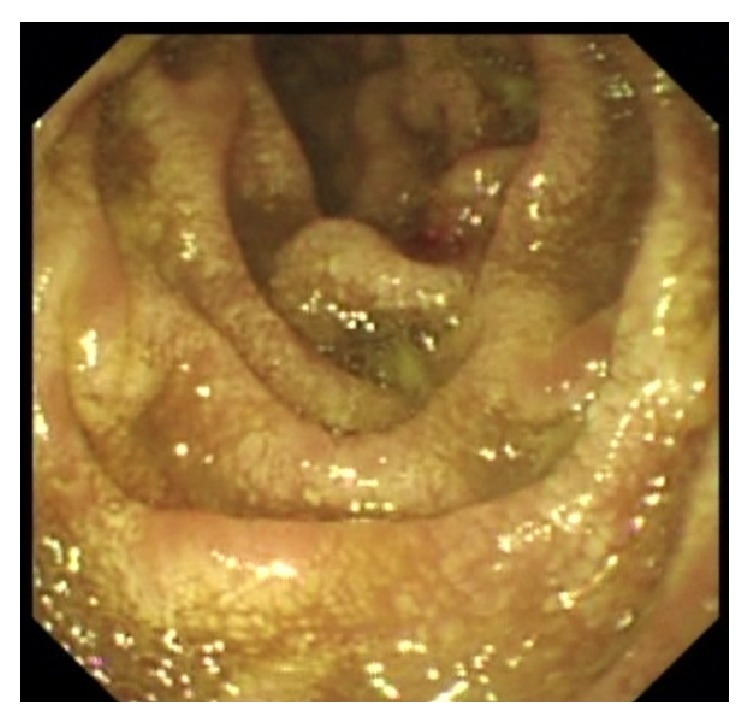
Second duodenum showing edematous mucosa with erosions and white villi.

**Figure 4:**
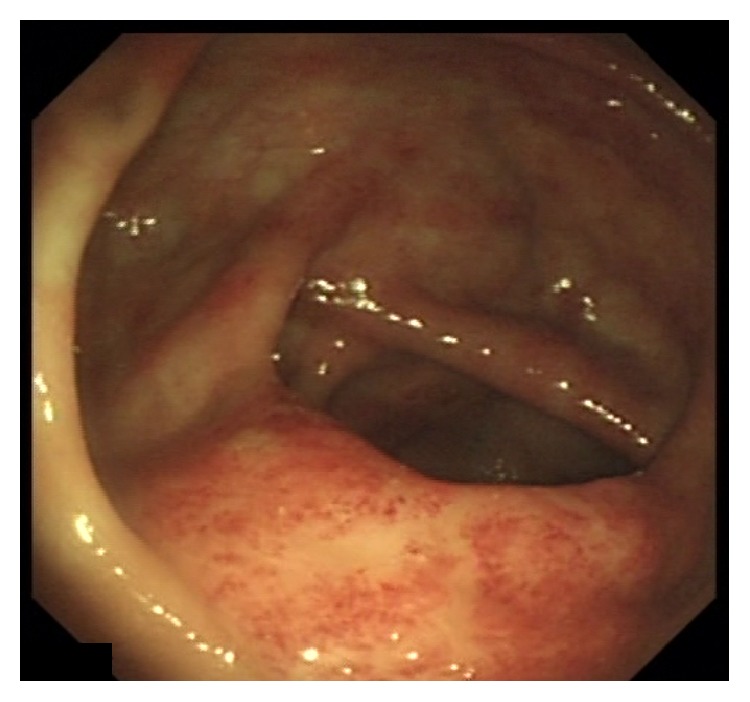
Cecal base showing patchy erythematous and petechial mucosa predominantly over the Ileocecal valve.

**Figure 5:**
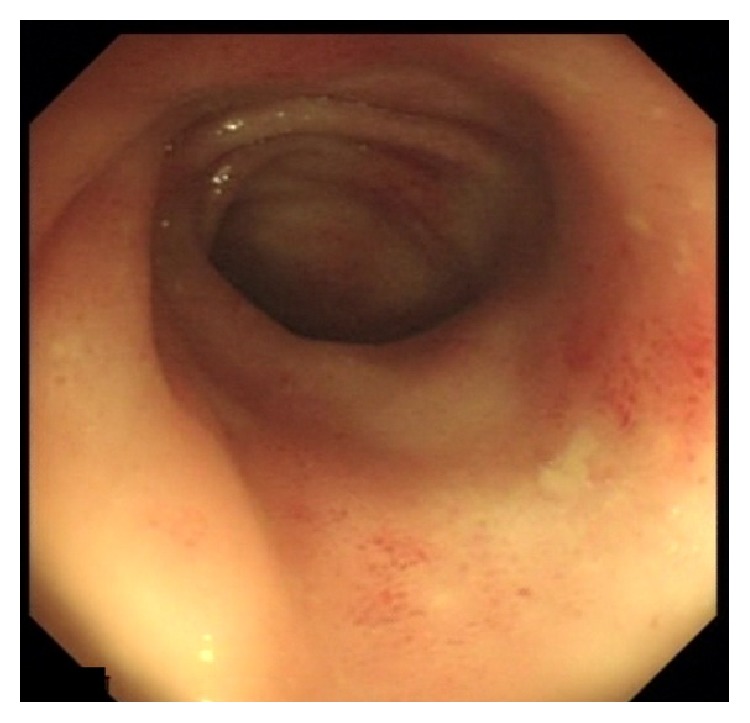
Terminal Ileum showing patchy erythematous lesions.

**Figure 6:**
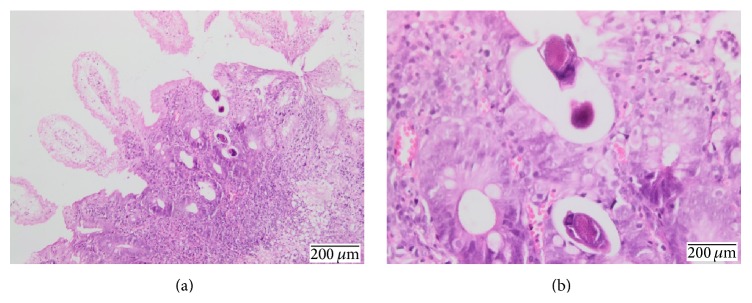
Duodenal biopsy showing preserved villi with dystrophic glands, multiple ulcerations, and eosinophilic infiltration.* Parasitic larvae* are also seen either superficial or encrusted into the mucosa compatible with strongyloidiasis.

In view of his unexplained eosinophilia associated with diarrhea and abdominal pain, together with his history of dyspnea and skin rash that flared up while on steroids and his brother's history of strongyloidiasis, we strongly suspected an underlying strongyloides infection. Thus, we requested strongyloides serology test and upper and lower endoscopies. Upper endoscopy showed severe edematous bulboduodenitis with areas of erosions and whitish villi (Figures 2 and 3) and lower endoscopy showed multiple patchy erythematous lesions separated by areas of normal mucosa that appeared all over the colon, predominantly in the cecum, and in the ileum (Figures 4 and 5). Duodenal biopsy showed severe erosive duodenitis with eosinophilic infiltration and strongyloides larvae (Figures 6(a) and 6(b)). Colonic and ileal biopsies showed severe eosinophilic inflammatory changes without parasitic detection. Serology test showed antistrongyloides antibody titer of 4.5 (normal <1.2). So patient was diagnosed to have strongyloidiasis.

Ivermectin was started at a dose of 200 mcg/kg/day. Patient's diarrhea and abdominal pain disappeared within 3 days. However, patient was still having high eosinophil count after 1 week. Since then, we extended Ivermectin course to 2 weeks until disappearance of eosinophilia. Stool test was obtained 2 weeks after initiating treatment and turned out to be negative. Albumin level was progressively increasing to 3.1 g/dL in about 1 month.

## 3. Discussion

Strongyloidiasis is a helminthic disease caused by* Strongyloides stercoralis* (*S. stercoralis*), a soil transmitted nematode. It affects 30–100 million people globally [1] being hyperendemic (>5%) or endemic (1–5%) in many tropical and subtropical areas including Sub-Saharan Africa, Latin and South America, and South-East Asia and sporadic (<1%) in other temperate areas [2]. In the MENA region, its prevalence varies between countries and it was found to be more frequent in Israel (94%) and Sudan (3.7%) according to community based surveys and in Iraq (24.2%), Kuwait (16.3%), Saudi Arabia (12.5%), and Egypt (11.1%) according to hospital based surveys [3, 4]. In contrast, in Lebanon,* S. stercoralis* is still considered as a rare infection with a reported prevalence of 0.1–0.5% [5, 6] but such values are believed to underestimate the true frequency of the disease due to the low sensitivity of single fecal analysis commonly used in practice as well as the rarity of case reports and studies targeting specifically strongyloidiasis prevalence, in addition to the predominance of subclinical and poorly symptomatic disease manifestations.

*S. stercoralis* is acquired by contact with contaminated soil, human waste, or sewage. Hence, conditions such as poor hygiene and crowding, as well as some occupations such as farming and coal mining, may increase the risk of infection. Such factors exist in some Lebanese populations, notably in the Northern Province, the hometown of our patient and his brother, where previous studies found a higher rate of parasitosis in general [6].

The filariform larvae, infectious form of the parasite, invade the skin of the affected individuals through which they migrate to the lungs either by hematogeneous or lymphatic spread. Then, they ascend up in the tracheobronchial tree until being swallowed into the GI tract where they mature. The adult female produces eggs that develop into noninfectious rhabditiform larvae. These larvae may mature again into infectious form resulting in a cycle of autoinfection, which enables the parasite to persist for decades after initial infection. However, any state of immunosuppression, most importantly steroid use [7], or other immunosuppressive drugs use, malignancies, AIDS, HTLV-1 infection, advanced age, or malnutrition may increase the parasite burden and accelerate the autoinfectious process inducing a hyperinfection syndrome which might develop into severe disseminated disease thereafter [8]. In our patient, advanced age and prednisone therapy precipitated the infectious flare up.

In acute infection, patients may exhibit a local skin rash followed by dyspnea and cough due to larvae migration into the lungs. During chronic autoinfection, patients may remain asymptomatic or may experience mild waxing and waning gastrointestinal, respiratory, or dermatologic symptoms with peripheral eosinophilia. Hyperinfection syndrome may have a wide range of clinical manifestations that increase in severity in parallel with the parasitic dissemination rate. Gastrointestinal symptoms are common including diarrhea, abdominal pain, and vomiting. Prolonged malabsorption and protein loosing enteropathy may also be present leading to severe hypoalbuminemia and refractory peripheral edema [9]. Pulmonary manifestations may include dyspnea and cough with lung infiltrates mimicking Loeffler Syndrome or even ARDS in severe cases [10, 11]. Skin involvement may present as pruritic rash that has sometimes a pathognomonic migratory serpiginous aspect termed “larva currens.” More severe disseminated disease may involve the CNS, the pancreas, the liver, and the heart, and it may be associated with Gram negative septicemia secondary to simultaneous bacterial seeding through the GI tract [8].

Our patient's hyperinfective syndrome was manifested clinically by gastrointestinal involvement including protein loosing enteropathy with diarrhea and abdominal pain in addition to skin involvement. Whether respiratory symptoms were related to strongyloidiasis or to another infection and pesticides inhalation is uncertain, but the improvement of dyspnea and chest infiltrate while on steroids and the absence of larvae on BAL examination argued against pulmonary strongyloidiasis manifestations.

Peripheral eosinophilia is a strong indicator of the infection in high risk population [12] but it is not sufficiently sensitive to be used as a sole screening test in chronic uncomplicated infection [13] due to its intermittent presence. On the other hand, it might be absent in disseminated disease especially in patients under steroid therapy [14]. Eosinophilia was documented in our patient 5 months before developing the hyperinfection syndrome but it was underappreciated at that time due to the low suspicion index of infection in our population.

Strongyloides larvae are generally passed in stools one month after initial infection. Higher parasitic load is observed during hyperinfection and disseminated disease where larvae detection by stool, sputum, bronchoalveolar lavage, and skin lesions examination is highly sensitive [15]. However, in chronic disease, single direct fecal examination only detects about 30% of infections [16]. Repeated stool test increases such sensitivity that may reach about 100% when 7 samples are used [17]. Other methods such as Formol-Ether Concentration Technique (FECT), the Baermann method, the Harada-Mori filter paper technique, and agar plate culture (APC) were suggested to improve fecal larvae detection rate. Of those, the APC technique is the most suitable having 89% sensitivity [18].

Serologic tests are also useful for strongyloidiasis diagnosis as was shown in our case. Enzyme-Linked Immuno-Sorbent Assay (ELISA) method is highly sensitive for antibody detection [19] but it has a low predictive value in high risk population due to the possible cross reactivity with other helminthes. A newer recombinant antigen based serologic method, the luciferase immunoprecipitation system (LIPS), decreased such confounding factor and improved serologic test reliability [20].

Enterotest or String test is also sensitive for larvae detection but is cumbersome for patients [21]. Molecular testing using PCR for DNA detection on stools samples also appears efficient [22].

Gastrointestinal endoscopy with biopsies may also play a role in the diagnosis. Many endoscopic features were described in the literature including edema and erythematous spots in the duodenum as well as loss of vascular pattern and ulcerations with patchy erythema in the colon but none are pathognomonic for strongyloidiasis [23]. Our reported duodenal endoscopic findings were similar to those reported by Kishimoto et al. study [24] concerning edematous mucosa and notably the white villi which may be a good endoscopic marker for strongyloidiasis in endemic regions. As* S. stercoralis* colonizes the duodenum, duodenal biopsies are the most accurate for larvae detection [23], and this finding was also illustrated in our case where only duodenal specimen showed the strongyloides larvae.

Uncomplicated infection is treated preferably with Ivermectin 200 mcg/kg given for 2 doses (over either 2 consecutive days or 2 weeks apart). Albendazole, Mebendazole, and Thiabendazole are possible alternatives. In hyperinfection syndrome and disseminated disease, same therapeutic modalities and notably Ivermectin can also be used but the appropriate duration of treatment still not well determined. It was suggested to give Ivermectin until getting symptomatic improvement with negative test for larvae for at least 2 weeks (duration of autoinfectious cycle) [25].

Posttreatment eradication should be confirmed to prevent further autoinfectious cycles. Improved eosinophilia, repeated stools examination in 2 weeks, and decreased antibody titer after 6 to 12 months may be useful methods for monitoring in this setting [26, 27].

Prognosis of strongyloidiasis is generally good but mortality rate may be as high as 71% in disseminated disease [14] and it may increase with concomitant bacteremia and severe immunosuppression [28].

## 4. Conclusion

We presented a case of strongyloidiasis, which is one of the most neglected helminthic infections worldwide and particularly in Lebanon, where we possibly have some underappreciated endemic areas. Index of suspicion should be raised in certain circumstances including peripheral eosinophilia and unexplained intermittent diarrhea or asthma-like symptoms or skin rashes. As it may cause serious burden in certain subgroups, further investigations should focus on implementing appropriate control programs.

## References

[1] Olsen A., van Lieshout L., Marti H. (2009). Strongyloidiasis—the most neglected of the neglected tropical diseases?. *Transactions of the Royal Society of Tropical Medicine and Hygiene*.

[2] Paula F. M., Costa-Cruz J. M. (2011). Epidemiological aspects of strongyloidiasis in Brazil. *Parasitology*.

[3] Rokni M. B., Lotfy W. M., Hotez P. J., de Silva N. R., McDowell M. A., Rafati S. (2014). Soil-transmitted helminth (STH) infections in the MENA region. *Neglected Tropical Diseases—Middle East and North Africa*.

[4] Schär F., Trostdorf U., Giardina F. (2013). Strongyloides stercoralis: global distribution and risk factors. *PLoS Neglected Tropical Diseases*.

[5] Araj G. F., Musharraheh U. M., Haydar A., Ghawi A., Itani R., Saliba R. (2011). Trends and prevalence of intestinal parasites at a tertiary care center in Lebanon over a decade. *Journal Medical Libanais*.

[6] Saab B. R., Musharrafieh U., Nassar N. T., Khogali M., Araj G. F. (2004). Intestinal parasites among presumably healthy individuals in Lebanon. *Saudi Medical Journal*.

[7] Suvajdžić N., Kranjčić-Zec I., Jovanović V., Popović D., Čolović M. (1999). Fatal strongyloidosis following corticosteroid therapy in a patient with chronic idiopathic thrombocytopenia. *Haematologia*.

[8] Keise P. B., Nutman T. B. (2004). Strongyloides stercoralis in the immunocompromised population. *Clinical Microbiology Reviews*.

[9] Ho P. L., Luk W. K., Chan A. C. L., Yuen K. Y. (1997). Two cases of fatal strongyloidiasis in Hong Kong. *Pathology*.

[10] Ahmed S., Rashid S., Ammannagari N., Cheungpasitporn W. (2013). Chasing Eosinophilia in Loeffler's syndrome: a case of strongyloidiasis in upstate New York. *North American Journal of Medical Sciences*.

[11] Newberry A. M., Williams D. N., Stauffer W. M., Boulware D. R., Hendel-Paterson B. R., Walker P. F. (2005). Strongyloides hyperinfection presenting as acute respiratory failure and gram-negative sepsis. *Chest*.

[12] Román-Sánchez P., Pastor-Guzmán A., Moreno-Guillén S., Igual-Adell R., Suñer-Generoso S., Tornero-Estébanez C. (2003). High prevalence of Strongyloides stercoralis among farm workers on the Mediterranean coast of Spain: analysis of the predictive factors of infection in developed countries. *The American Journal of Tropical Medicine and Hygiene*.

[13] Sudarshi S., Stümpfle R., Armstrong M. (2003). Clinical presentation and diagnostic sensitivity of laboratory tests for *Strongyloides stercoralis* in travellers compared with immigrants in a non-endemic country. *Tropical Medicine and International Health*.

[14] Lam C. S., Tong M. K. H., Chan K. M., Siu Y. P. (2006). Disseminated strongyloidiasis: a retrospective study of clinical course and outcome. *European Journal of Clinical Microbiology and Infectious Diseases*.

[15] Buonfrate D., Requena-Mendez A., Angheben A. (2013). Severe strongyloidiasis: a systematic review of case reports. *BMC Infectious Diseases*.

[16] Siddiqui A. A., Berk S. L. (2001). Diagnosis of *Strongyloides stercoralis* infection. *Clinical Infectious Diseases*.

[17] Nielsen P. B., Mojon M. (1987). Improved diagnosis of strongyloides stercoralis by seven consecutive stool specimens. *Zentralblatt fur Bakteriologie Mikrobiologie und Hygiene A*.

[18] Polanco L. C., Gutiérrez L. A., Arias J. C. (2014). Diagnosis of strongyloides stercoralis infection: meta-analysis on evaluation of conventional parasitological methods (1980–2013). *Revista Española de Salud Pública*.

[19] Carroll S. M., Karthigasu K. T., Grove D. I. (1981). Serodiagnosis of human strongyloidiasis by an enzyme-linked immunosorbent assay. *Transactions of the Royal Society of Tropical Medicine and Hygiene*.

[20] Krolewiecki A. J., Ramanathan R., Fink V. (2010). Improved diagnosis of *Strongyloides stercoralis* using recombinant antigen-based serologies in a community-wide study in northern Argentina. *Clinical and Vaccine Immunology*.

[21] Goka A. K. J., Rolston D. D. K., Mathan V. I., Farthing M. J. G. (1990). Diagnosis of *Strongyloides* and hookworm infections: comparison of faecal and duodenal fluid microscopy. *Transactions of the Royal Society of Tropical Medicine and Hygiene*.

[22] Moghaddassani H., Mirhendi H., Hosseini M., Rokni M. B., Mowlavi G., Kia E. (2011). Molecular diagnosis of strongyloides stercoralis infection by PCR detection of specific DNA in human stool samples. *Iranian Journal of Parasitology*.

[23] Thompson B. F., Fry L. C., Wells C. D. (2004). The spectrum of GI strongyloidiasis: an endoscopic-pathologic study. *Gastrointestinal Endoscopy*.

[24] Kishimoto K., Hokama A., Hirata T. (2008). Endoscopic and histopathological study on the duodenum of *Strongyloides stercoralis* hyperinfection. *World Journal of Gastroenterology*.

[25] Mejia R., Nutman T. B. (2012). Screening, prevention, and treatment for hyperinfection syndrome and disseminated infections caused by *Strongyloides stercoralis*. *Current Opinion in Infectious Diseases*.

[26] Loutfy M. R., Wilson M., Keystone J. S., Kain K. C. (2002). Serology and eosinophil count in the diagnosis and management of strongyloidiasis in a non-endemic area. *American Journal of Tropical Medicine and Hygiene*.

[27] Montes M., Sawhney C., Barros N. (2010). Strongyloides stercoralis: there but not seen. *Current Opinion in Infectious Diseases*.

[28] El Masry H. Z., O'Donnell J. (2005). Fatal stongyloides hyperinfection in heart transplantation. *Journal of Heart and Lung Transplantation*.

